# Gold nanoparticle-labeled biosensor for rapid and sensitive detection of bacterial pathogens

**DOI:** 10.1186/s13036-015-0014-z

**Published:** 2015-10-02

**Authors:** Yun Wang, Evangelyn C. Alocilja

**Affiliations:** Department of Biosystems and Agricultural Engineering, Michigan State University, East Lansing, MI 48824 USA; Present address: Division of Food Processing Science and Technology, U. S. Food and Drug Administration, Bedford Park, IL 60501 USA

**Keywords:** *E. coli* O157:H7, Biosensor, Rapid detection, Nanoparticles, Magnetic separation, Electrochemical measurement, Antibodies

## Abstract

**Background:**

*Escherichia coli* O157:H7 is one of the major foodborne bacterial pathogens and also a biodefense agent. To ensure food safety and public health, it is very important to develop rapid methods for *E. coli* O157:H7 detection. In this study, we designed a nanoparticle-labeled biosensor for the rapid detection of *E. coli* O157:H7 in broth.

**Results:**

Magnetic nanoparticles (MNPs) were conjugated with monoclonal antibodies (Abs) to separate target *E. coli* O157:H7 cells from broth samples*.* Gold nanoparticles (AuNPs) were conjugated with polyclonal Abs, and were then introduced to the MNP-target complex to form a sandwich MNP-target-AuNP. By measuring the amount of AuNPs through an electrochemical method, the presence and the amount of the target bacteria were determined. Results showed a sensitivity of 10^1^ colony forming units per milliliter (cfu/ml) with a linear range of 10^1^–10^6^ cfu/ml.

**Conclusions:**

Compared to conventional culture plating methods, the biosensor reduced the detection time from 2 to 4 days to less than 1 hour with a simple target extraction method. The AuNP-labeled biosensor has potential applications in the rapid detection of infectious agents for public health, biodefense, and food/water safety.

## Background

Rapid detection of pathogenic bacteria is critical to public health, biodefense, and food/water safety. *Escherichia coli* O157:H7 is one of the major foodborne bacterial pathogens and also a biodefense agent. There were several outbreaks of *E. coli* O157:H7 in recent years that endangered public health [1–3]. Because conventional culture plating methods for *E. coli* O157:H7 take two to four days to obtain results, development of rapid detection methods for this organism is important. Biosensors are emerging technologies that have the potential for getting rapid results and that can be employed in the field. There are many biosensor configurations and approaches that are in the research and design stage. These configurations include antibody-based systems [4–8], enzyme-based detection [9, 10] and DNA-based sensors [11, 12]. In addition to speed, biosensors have the potential to generate highly sensitive results. This is especially critical as many bacterial infections could be caused by as low as 10 organisms [13].

The application of nanomaterials in biosensors, such as nanoparticles with optical, electronic and magnetic properties, has drawn interest. Because of their unique characteristics, nanoparticles have been used to enhance sensor sensitivity either by increasing the capture efficiency of the target molecules or by utilizing the optical and electronic properties of the nanostructures to amplify signals. Magnetic nanoparticles were employed for separating targets for bacterial detection [6, 14, 15]. Gold nanoparticles (AuNPs) were used for signal amplification [15, 16]. Polymeric nanoparticles were also introduced for signal amplification [6, 17].

In this paper, we developed an electrochemical biosensor using antibody-modified nanoparticles for the detection of *E. coli* O157:H7. Two novel nanoparticles were utilized in the biosensor design: 1) polymer-coated magnetic nanoparticles (MNPs) to separate the target bacteria from the sample matrix and carbohydrate-capped AuNPs to label the separated target by forming a sandwich structure and generate the signal. The signal of AuNPs for the corresponding target was measured by differential pulse voltammetry (DPV) on a screen printed carbon electrode (SPCE) chip. The biosensor enabled rapid pathogen detection in 45 min from sample preparation to final readout of results.

## Results and discussion

### Magnetic separation of target *E. coli* O157:H7

**Fig. 1:**
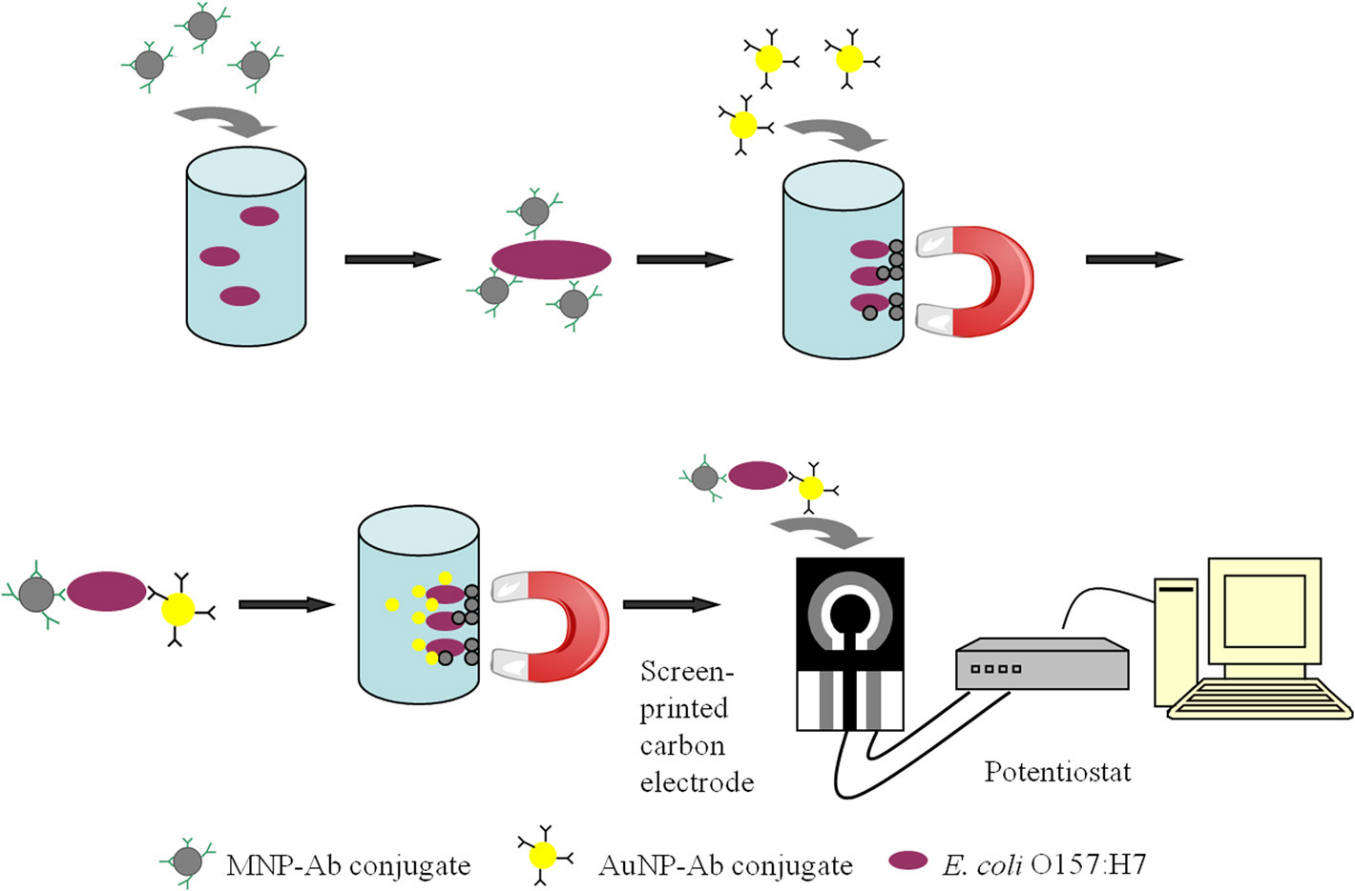
Schematic of the gold nanoparticle (AuNP)-labeled biosensor. Target cells in a sample were captured by magnetic nanoparticle (MNP)-antibody (Ab) conjugates and separated by a magnet. Then the cells were labeled with AuNPs. The MNP-Ab-cell-Ab-AuNP complexes were transferred onto a screen printed carbon electrode that is a chip connected to a potentiostat for electrochemical measurement

**Fig. 2:**
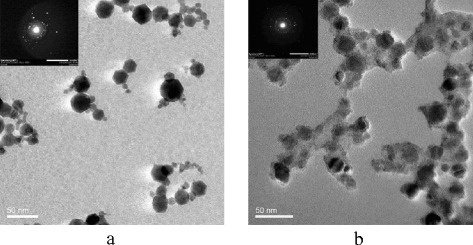
Polyaniline (PANI)-coated magnetic nanoparticles (MNPs). Transmission electron microscopy (TEM) images of: (**a**) Fe_2_O_3_ core; (**b**) PANI-coated MNPs. The insets show the electron diffraction patterns of the nanoparticles [18]. Used with permission from Biosensors & Bioelectronics

*E. coli* O157:H7 cells were magnetically captured as shown in Fig. 1. We modified the Fe_2_O_3_ nanoparticles with polyaniline (PANI) for direct immobilization of anti-*E. coli* O157:H7 antibody (Ab). Figure 2 presents the transmission electron microscopy (TEM) images of the Fe_2_O_3_ nanoparticle core (Fig. 2a) and the PANI-coated MNPs (Fig. 2b) [18]. Figure 2a reveals that the average diameter of the Fe_2_O_3_ nanoparticle core is 20 nm, while Fig. 2b shows that the PANI-coated MNPs have diameters ranging from 50 to 100 nm. The increase of the diameter was due to the formation of PANI around the Fe_2_O_3_ core. According to the insets, the electron diffraction pattern in Fig. 2a exhibited a typical maghemite (γ-Fe_2_O_3_) nanoparticle structure [19]. In Fig. 2b, the electron diffraction pattern shows a set of rings which are typical for PANI [20], noted that it has less bright spots than in Fig. 2a. This pattern also indicates the coating of Fe_2_O_3_ core by PANI.

**Fig. 3:**
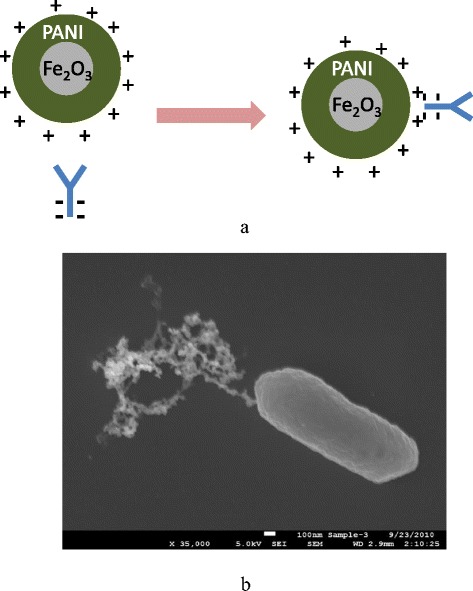
**a** Schematic of the conjugation of Polyaniline (PANI)-coated magnetic nanoparticles (MNPs) and antibody; and (**b**) Scanning electron microscopy (SEM) image of an antibody-conjugated MNP bound to an *E.coli* O157:H7 cell [6]; used with permission from International Journal of Food Safety, Nutrition and Public Health (Inderscience retains copyright of the original article and figure)

Electrostatic interaction has been used to modify the PANI-coated MNPs with antibody. The interaction between the negatively charged Fc fragment of antibody molecules and the positively charged PANI contributes to the conjugation [18]. Figure 3a shows a schematic of the interaction between the PANI-coated MNPs and the antibody.

The MNP-Ab was used to magnetically separate the target from the sample through the antibody-antigen interaction. The scanning electron microscopy (SEM, Fig. 3b) confirms the capture of the target [6]. In this image, a rod-shaped *E. coli* O157:H7 cell is bound to MNP-Ab conjugate.

### AuNP-labeled biosensor for detection

**Fig. 4:**
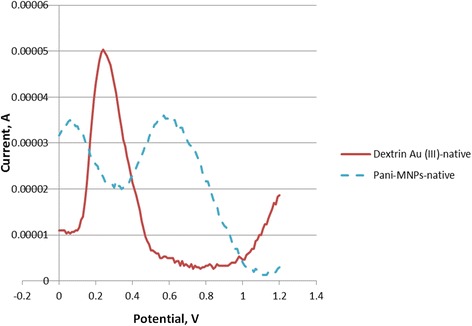
Differential pulse voltammetric sensorgrams of the gold nanoparticle (AuNPs) and Polyaniline (PANI)-coated magnetic nanoparticles (MNPs). AuNPs show a current peak at 0.3 V and MNPs show a current peak at 0.58 V

**Fig. 5:**
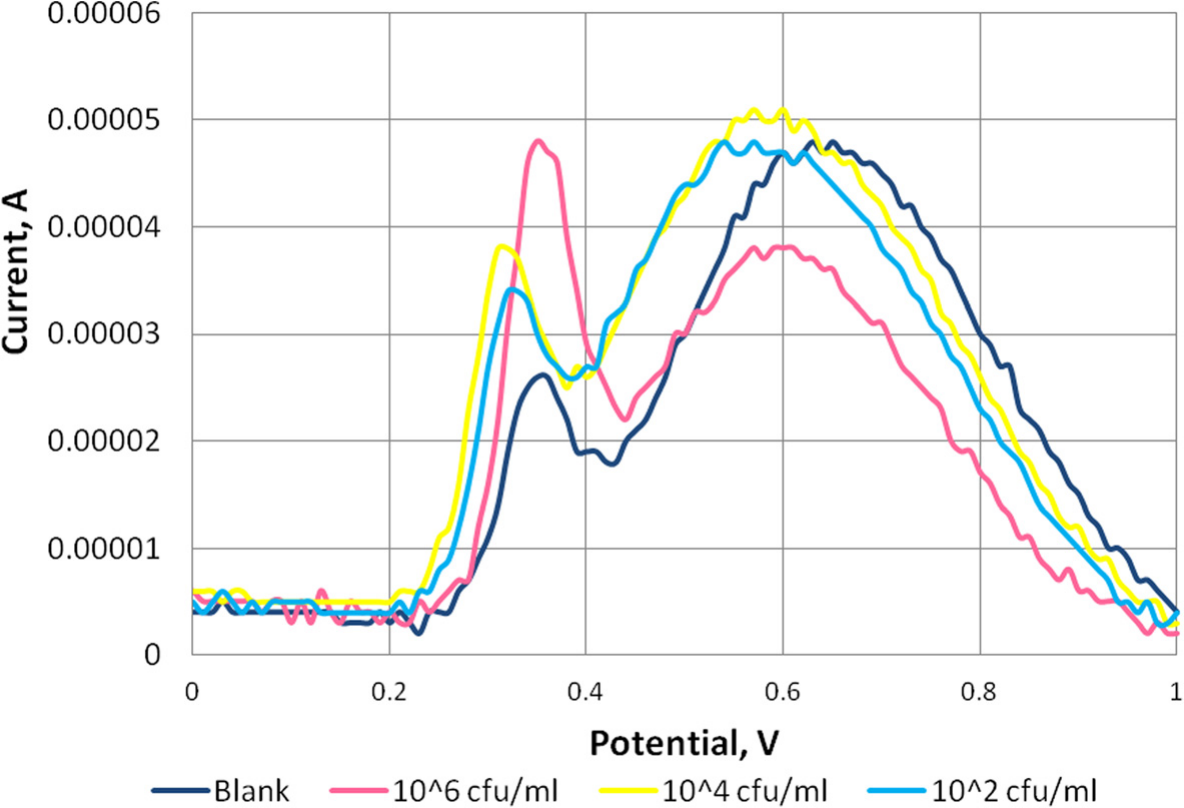
Sensorgram of gold nanoparticle (AuNP)-labeled biosensor for *E. coli* O157:H7 detection. Peak current for AuNP at around 0.3 V increases with increasing cell concentrations

Figure 4 shows two typical sensorgrams of native AuNPs and native MNPs. Current peak for AuNPs is at 0.3 V and MNPs is at 0.58 V. Figure 5 shows typical DPV sensorgrams for the detection of *E. coli* O157:H7 in different cell concentrations (10^2^, 10^4^, and 10^6^ colony forming units per milliliter, cfu/ml). The sensorgrams show a wide curve that seems to include both AuNPs and MNPs. For the analysis, peak current to the left (representing AuNPs) around 0.3 V was chosen for signal reporting. As shown in the graph, peak current for AuNPs increased with increasing cell concentration. Figure 5 confirms the formation of the MNP-cell-AuNP complex. The amount of target cells detected was proportional to the amount of AuNPs.

**Fig. 6:**
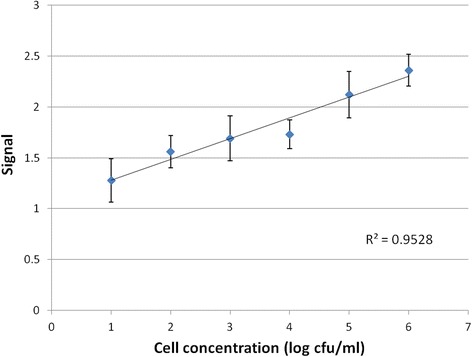
Peak current vs. cell concentration of the gold nanoparticle (AuNP)-labeled biosensor for *E. coli* O157:H7 detection. The signal shows a linear relationship between 10^1^ to 10^6^ cfu/ml

A linear relationship between signal to noise ratio (SNR) and cell concentration is shown in Fig. 6, with an R^2^ value of 0.953. The SNR was calculated as the peak current of AuNPs for each concentration divided by the current at 0.3 V of the blank, which was used as the signal of this biosensor. The SNR for 10^1^, 10^2^, 10^3^, 10^4^, 10^5^ and 10^6^ cfu/ml are 1.28, 1.56, 1.69, 1.73, 2.12 and 2.36, respectively. A statistical analysis using *t* test was conducted and the results are presented in Table 1. As shown, cell concentration at 10^1^ cfu/ml has a *P* value of 0.0546 (which is close to critical value of *P* = 0.05). The *P* value for other concentrations shows that the samples are significantly different from the blank. Therefore, the lowest cell concentration is weakly 10^1^ cfu/ml and strongly at 10^2^ cfu/ml. These results verify that MNP-Ab-cell-Ab-AuNP is an effective approach to highly sensitive detection. The specificity of the biosensor, which largely depends on the monoclonal antibody used in magnetic separation, was evaluated in another study by our lab [21]. An inclusivity of 94 % and an exclusivity of 69 % was obtained [21]. The inclusivity was calculated as the number of positive tests identified by the antibody divided by the actual total positive test number. The exclusivity was calculated as the number of negative tests identified divided by the actual total negative test number. The specificity of the monoclonal and polyclonal Abs-based sandwich configuration was also verified in our previous study [8].

**Table 1:** Statistical analysis comparing samples and blank (*t* test)

Concentration (cfu/ml)	10^1^	10^2^	10^3^	10^4^	10^5^	10^6^
*P* value^*^	0.0546	0.0212	0.0044	0.0054	0.0079	0.0007

^*^ *P* value was calculated by a one-tailed paired *t* test. The critical value, *P* = 0.05

## Conclusions

A AuNP-labeled biosensor has been developed for the sensitive and rapid detection of *E. coli* O157: H7. The lower limit of detection is at 10^1^ cfu/ml with a dynamic range of 10^1^ to 10^6^ cfu/ml. Target extraction and detection are completed within 45 min. Furthermore, sample preparation requires a simple magnet without special equipment. Due to its simplicity and rapid results, this biosensor has the potential for in-field use in quickly screening bacterial contamination in food products (e.g. ground beef, vegetables, and juices) and water systems. The biosensor is also a promising technology for point-of-care screening of diseases and environmental determination of harmful biological agents.

## Materials and methods

### Reagents and materials

Two kinds of nanoparticles were synthesized: MNPs and AuNPs. Aniline, iron (III) oxide nanopowder, ammonium persulfate, methanol, and diethyl ether (Sigma-Aldrich, St. Louis, MO) were used for the synthesis of the MNPs. Gold (III) chloride trihydrate (Aldrich, MO) and dextrin (Fluka, MO) were used for the synthesis of AuNPs under alkaline conditions [22].

MNPs were functionalized with monoclonal anti-*E. coli* O157:H7 antibody obtained from Meridian Life Science, Inc (Saco, ME). AuNPs were conjugated with polyclonal anti- *E. coli* O157:H7 antibody from Meridian Life Science, Inc (Saco, ME). Protein A from *Staphylococcus aureus* (Sigma-Aldrich, St. Louis, MO) was used as the linkage agent for AuNP and antibody conjugation.

Triton-X100, phosphate buffered saline (PBS), casein, bovine serum albumin (BSA) and sodium phosphate (dibasic and monobasic) were obtained from Sigma-Aldrich (St. Louis, MO). PBS buffer (0.01 M and 0.1 M, pH 7.4), 0.01 M PBS buffer with 0.05 % (w/v) Triton-X100, phosphate buffer (0.1 M sodium phosphate, pH 7.4), 0.01 M PBS buffer with 0.01 % casein, 0.01 M PBS buffer with 0.1 % (w/v) BSA were prepared with deionized water from Millipore Direct-Q system. PBS buffer and phosphate buffer were used in preparing nanoparticle-Ab conjugates and in washing. PBS buffer with casein or BSA was used to block nanoparticle surface against nonspecific binding. PBS buffer with Triton-X100 was used for washing off unbound or nonspecifically bound reactants after capture.

### Bacterial culture

*E. coli* O157:H7 Sakai strain was obtained from the Nano-Biosensors Lab collection at Michigan State University. The colonies from frozen (stored at –70 °C) culture were grown on trypticase soy agar (BD Biosciences, MD) plates. A single colony was isolated and inoculated in tryptic soy broth (BD Biosciences, MD) and grown overnight at 37 °C. One milliliter of the liquid culture was transferred to another tube of tryptic soy broth and incubated overnight at 37 °C. One milliliter of this liquid culture was transferred to a new tube of broth and incubated at 37 °C for 6 h before each experiment. The serial dilutions of bacterial culture were prepared using 0.1 % (w/v) peptone water (Fluka-Biochemika, Switzerland) before each experiment. Viable cells were enumerated by microbial plating on Sorbitol MacConkey agar (SMAC, BD Biosciences, MD).

### Apparatus

Electrochemical measurement was performed with a potentiostat/galvanostat (263A, Princeton Applied Research, MA) with a software operating system (PowerSuite, Princeton Applied Research, MA) on a computer connected to the potentiostat. The measurement was performed by introducing each sample onto a SPCE chip (Gwent Inc. England). The SPCE chip consisted of a working carbon electrode and a counter and reference silver/silver chloride electrode. One hundred microliters of each sample were introduced to the electrode area on the SPCE chip.

### Synthesis of nanoparticles

PANI coated MNPs were synthesized according to our method [6]. Fifty milliliters of 1 M hydrochloric acid, 10 ml of water and 0.4 ml of aniline monomer were mixed in a flask, and then 0.65 g of iron (III) oxide nanopowder were added to the solution to maintain a final γ-Fe_2_O_3_: aniline weight ratio of 1: 0.6. The mixture was put in a beaker filled with ice and sonicated for 1 h. The solution was stirred while it was still on ice. During the stirring, ammonium persulfate (1 g of ammonium persulfate in 20 ml deionized water) was added to the solution slowly for 30 min. The solution was stirred for another 1.5 h. After the reaction, the solution was filtered using 2.5 μm filter paper and washed with 20 % methanol. Hydrochloric acid (1 M) was used to wash until the filtrate became clear, followed by washing with 10 ml of 20 % methanol. The filtrate was filtered again using a 1.2 μm filter paper, and 10 ml of 20 % methanol solution was added to the filter. The hydrochloric acid and methanol wash was repeated. The nanoparticles on the filter paper were left under a fume hood to dry for 24 h at room temperature and stored in a vacuum desiccator after drying.

AuNPs were synthesized under alkaline conditions following the approach published by Anderson et al [22]. Briefly, 20 ml of dextrin stock solution (25 g/l) and 20 ml of sterile water were mixed in a 50 ml sterile orange cap tube (disposable). Five milliliters of HAuCl_4_ stock solution (8 g/ml) were then added, and the pH of the solution was adjusted to 9 with sterile 10 % (w/v) Na_2_CO_3_ solution. The final volume was brought to 50 ml with pH 9 water. The reaction was carried out by incubating the solution in a sterile flask in the dark at 50 °C with continuous shaking (100 rpm) for 6 h. A red solution was obtained at the end of the reaction.

### Functionalization of nanoparticles

MNPs were functionalized with a monoclonal anti-*E. coli* O157:H7 antibody [6]. MNPs (2.5 mg) were suspended in 150 μl of 0.1 M phosphate buffer, and sonicated for 15 min. Monoclonal anti-*E. coli* O157:H7 antibody (2.5 mg/ml, 100 μl) was added to the suspension, and hybridized on tube rotator for 5 min. Twenty five microliters of PBS buffer (0.1 M) were added. Then the conjugation was carried on for 55 min on the tube rotator. The MNPs were separated from the solution by magnetic separation, and blocked by adding 250 μl of 0.1 M tris buffer with 0.01 % casein and incubated for 5 min. This step was repeated three times, and the suspension was put on tube rotator for 1 h to hybridize. Finally, the MNPs were magnetically separated and resuspended in 2.5 ml of 0.1 M phosphate buffer. The MNP-Ab conjugate was stored at 4 °C before use.

AuNPs were conjugated with a polyclonal anti-*E. coli* O157:H7 antibody through protein A linkage. Two hundred microliters of 1:2 diluted suspension of AuNPs in water were put into a 2 ml microcentrifuge tube and sonicated for 10 min. Then the suspension was centrifuged for 6 min at 13,000 rpm. The supernatant was removed after centrifugation. To modify the surface of the AuNPs, protein A (0.25 mg/ml) in 200 μl of 0.01 M PBS buffer was used to resuspend the AuNPs. The conjugation was conducted by rotating the mixture for 1 h. The modified AuNPs were separated from the suspension by centrifugation for 6 min at 13,000 rpm. The nanoparticles were washed by adding 200 μl of 0.01 M PBS buffer and centrifuged. After removing the supernatant, 100 μl of 1 mg/ml antibody and 100 μl of 0.01 M PBS buffer were added to the tube and mixed for 60 min by rotating. After separating the AuNP-antibody (AuNP-Ab) conjugates from the supernatant, 200 μl of PBS buffer with 0.1 % (w/v) BSA were added to the tube. The mixture was rotated for 30 min. Finally, the AuNP-Ab conjugates were separated from the suspension by centrifugation, and the final suspension of the conjugates in 200 μl PBS buffer with BSA was stored at 4 °C.

### Detection of target pathogenic bacteria

Detection of the target pathogen is presented in Fig. 1. Blank control for the tests was peptone water in the same volume as the sample. Firstly, 400 μl of 0.01 M PBS buffer, 50 μl of cell dilution (or peptone water for the blank) and 50 μl of MNP-Ab conjugates were combined in a 2 ml sterile tube. After 15 min hybridization, PBS buffer (55 μl, 0.01 M) with 0.1 % BSA was added to the mixture as a blocking agent. Then, the MNP-*E. coli* complexes were magnetically separated from the solution and resuspended in 450 μl of 0.01 M PBS buffer. Secondly, 50 μl of the AuNP-Ab conjugates were introduced to the system, followed by 15 min hybridization. After washing the complexes once with 0.01 M PBS buffer, the complexes were resuspended in 500 μl of PBS buffer with 0.05 % Triton-X100, and let stand for 3 min. Finally, the complexes were magnetically separated from the buffer and resuspended in 500 μl of 0.01 M PBS buffer. One hundred microliters of the suspension were plated on SMAC for cell counting. The rest of the complexes were magnetically separated from the supernatant (400 μl).

### Electrochemical measurement

The target bacteria were detected by measuring the electrochemical signal of AuNPs. Each sample from the last section (complexes magnetically separated from supernatant) in 100 μl 1 M hydrochloric acid was introduced to the SPCE chip. An oxidation potential of 1.4 V vs. Ag/AgCl was applied to the working electrode. After oxidation, a differential pulse voltammetric (DPV) measurement was performed. The scan was from 1.5 V to −1.5 V. The potential and currents were recorded. All measurements were performed at room temperature. Each sample was measured three times. At least three samples of each concentration of bacteria were tested.

## Abbreviations

AuNPs: Gold nanoparticles
MNPs: Magnetic nanoparticles
DPV: Differential pulse voltammetry
SPCE: Screen printed carbon electrode
TEM: Transmission electron microscopy
PANI: Polyaniline
Ab: Antibody
SEM: Scanning electron microscopy
SNR: Signal to noise ratio
PBS: Phosphate buffered saline
BSA: Bovine serum albumin
SMAC: Sorbitol MacConkey agar
